# Causal associations and shared genetic etiology of neurodegenerative diseases with epigenetic aging and human longevity

**DOI:** 10.1111/acel.14271

**Published:** 2024-09-19

**Authors:** Yu Guo, Guojuan Ma, Yukai Wang, Tingyan Lin, Yang Hu, Tianyi Zang

**Affiliations:** ^1^ School of Computer Science and Technology Harbin Institute of Technology Harbin China; ^2^ Beidahuang Industry Group General Hospital Harbin China

**Keywords:** causal relationship, epigenetic age acceleration, epigenetic clock, longevity, neurodegenerative disorders, shared genetic etology

## Abstract

The causative mechanisms underlying the genetic relationships of neurodegenerative diseases with epigenetic aging and human longevity remain obscure. We aimed to detect causal associations and shared genetic etiology of neurodegenerative diseases with epigenetic aging and human longevity. We obtained large‐scale genome‐wide association study summary statistics data for four measures of epigenetic age (GrimAge, PhenoAge, IEAA, and HannumAge) (*N* = 34,710), multivariate longevity (healthspan, lifespan, and exceptional longevity) (*N* = 1,349,462), and for multiple neurodegenerative diseases (*N* = 6618–482,730), including Lewy body dementia, Alzheimer's disease (AD), Parkinson's disease, amyotrophic lateral sclerosis, and multiple sclerosis. Main analyses were conducted using multiplicative random effects inverse‐variance weighted Mendelian randomization (MR), and conditional/conjunctional false discovery rate (cond/conjFDR) approach. Shared genomic loci were functionally characterized to gain biological understanding. Evidence showed that AD patients had 0.309 year less in exceptional longevity (IVW beta = −0.309, 95% CI: −0.38 to −0.24, *p* = 1.51E‐19). We also observed suggestively significant causal evidence between AD and GrimAge age acceleration (IVW beta = −0.10, 95% CI: −0.188 to −0.013, *p* = 0.02). Following the discovery of polygenic overlap, we identified rs78143120 as shared genomic locus between AD and GrimAge age acceleration, and rs12691088 between AD and exceptional longevity. Among these loci, rs78143120 was novel for AD. In conclusion, we observed that only AD had causal effects on epigenetic aging and human longevity, while other neurodegenerative diseases did not. The genetic overlap between them, with mixed effect directions, suggested complex shared genetic etiology and molecular mechanisms.

## Graphical abstract

Alzheimer's disease (AD) patients may slow down exceptional longevity and GrimAge age acceleration. We identified shared genomic loci, including a novel locus (rs78143120), for AD and GrmiAge age acceleration, and another locus (rs12691088) was associated with AD and exceptional longevity. Mixed effect directions indicated complex shared genetic etiology and mechanisms.

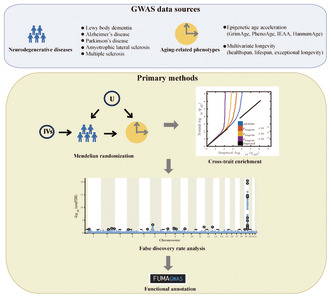

**Abbreviations**
AD: Alzheimer's disease
ALS: amyotrophic lateral sclerosis
cML‐MA: constrained maximum likelihood and model average‐based MR method
cond/conjFDR: conditional/conjunctional false discovery rate
EAA: epigenetic age acceleration
eQTL: expression quantitative trait locus
GTEx: Genotype Tissue Expression
GWAS: genome‐wide association study
IGAP: International Genomics of Alzheimer's Project
IMSGC: International Multiple Sclerosis Genetics Consortium
IPDGC: International Parkinson's Disease Genomics Consortium
LBD: Lewy body dementia
MHC: major histocompatibility complex
MR: Mendelian randomization
MS: multiple sclerosis
PD: Parkinson's disease
RCT: randomized controlled trial

## 1 INTRODUCTION

Both neurodegenerative diseases and human aging are time‐dependent disease and accompany each other (Farooqui & Farooqui, 2009; Kesidou et al., 2023; Villoslada et al., 2020). Aging is known to be a major risk factor for neurodegenerative diseases, including Alzheimer's disease (AD), Parkinson's disease (PD), and Lewy body dementia (LBD) (Hou et al., 2019; Hu et al., 2020, 2021). It suggests a possible genetic association and shared biological mechanisms between neurodegenerative diseases and human aging. However, to the best of our knowledge, few studies have researched whether neurodegenerative diseases contribute to aging. Therefore, it is both challenging and motivating us to investigate whether neurodegenerative diseases accelerate or slow aging.

Worldwide, approximately 44 million people lived with dementia and 6.1 million with PD in 2016, and the incidence of AD and PD is expected to more than double by 2050 (GBD 2016 Dementia Collaborators, 2019; GBD 2016 Parkinson's Disease Collaborators, 2018; Yang et al., 2023). Neurodegenerative diseases have become a major public health problem, imposing an enormous social and economic burden (Behr et al., 2023). Moreover, patients with these neurodegenerative diseases experience a severe impact on their quality of life and healthy lifespan (Hou et al., 2019). Therefore, uncovering the causal relationship and shared genetic etiology of neurodegenerative diseases and human longevity may help identify people at risk and promote healthy aging. Totally, it is necessary to investigate the specific genetic associations between neurodegenerative diseases and human aging, and longevity.

Aging phenotypes have been employed to evaluate the level of aging and applied in genetic studies (Timmers et al., 2020; Zhang & Liu, 2023). Our study focused on epigenetic clocks (GrimAge, PhenoAge, IEAA, and HannumAge) and human longevity phenotypes, including healthspan, parental lifespan (referred to hereafter as lifespan), and exceptional longevity. Several recent studies have pinpointed measures of epigenetic age, also known as epigenetic clocks, as the best predictor of biological age currently available (Horvath & Raj, 2018; Jylhava et al., 2017). Epigenetic age may not be consistent with the chronological age of the individual, biologically younger or older than the chronological age, known as epigenetic age acceleration (EAA) (Liu et al., 2020). Different epigenetic clocks capture different characteristics of the biological aging process. HannumAge (Hannum et al., 2013) and Intrinsic HorvathAge (Horvath, 2013) are “first‐generation” epigenetic clocks. HannumAge utilizes 71 CpG sites based on Illumina 450k array and is best with whole blood samples (Hannum et al., 2013). Intrinsic HorvathAge is a multi‐tissue age predictor trained on 353 CpG sites based on Illumina 27k array, allowing one to estimate the age of DNA methylation for most tissues and cell types (Horvath, 2013). The “second‐generation” epigenetic clocks include PhenoAge (Levine et al., 2018) and GrimAge (Lu et al., 2019). PhenoAge is trained on 513 CpG sites and developed using a two‐stage procedure (McCrory et al., 2021). Besides, PhenoAge surpasses “first‐generation” clocks in predicting many age‐related disorders and longevity (McCrory et al., 2021). GrimAge combined data from 1030 CpGs associated with smoking pack years and seven plasma proteins (Lu et al., 2019). Though different samples, methods, and characteristics, all epigenetic clocks have been validated for their ability to assess epigenetic age accurately (Horvath & Raj, 2018; Liu et al., 2020).

We perceived some limitations of the available studies on the genetic association of neurodegenerative diseases with EAA and longevity. First, studies have indicated that epigenetic clocks were involved in some neurodegenerative diseases, such as AD (Hu et al., 2023; Levine et al., 2015; Lu et al., 2017), PD (Jakubowski & Labrie, 2017), and amyotrophic lateral sclerosis (ALS) (Zhang et al., 2016). However, to the best of our knowledge, no studies have revealed genetic associations of MS and LBD with EAA and longevity. The unidentified causal effects and genetic overlap between them prompted us to further explore. Next, observational studies may run the risk of not controlling for confounding factors (e.g., chronic inflammation (Lee, 1983) and drug intake; Fleming et al., 2001), thereby obscuring the real outcome. Randomized controlled trials (RCTs) are the gold standard for demonstrating a causal relationship between medical‐related exposures as and outcomes. However, RCTs are always expensive and long in duration. It is also difficult to ensure that the studies are ethical and can be extended to other populations (Evans & Davey Smith, 2015). Notably, Mendelian randomization (MR) analysis has been widely applied for causal inferences in epidemiology, systems biology, pharmacogenomics, and other fields with the advantage of overcoming unmeasured confusions and low cost.

**FIGURE 1:**
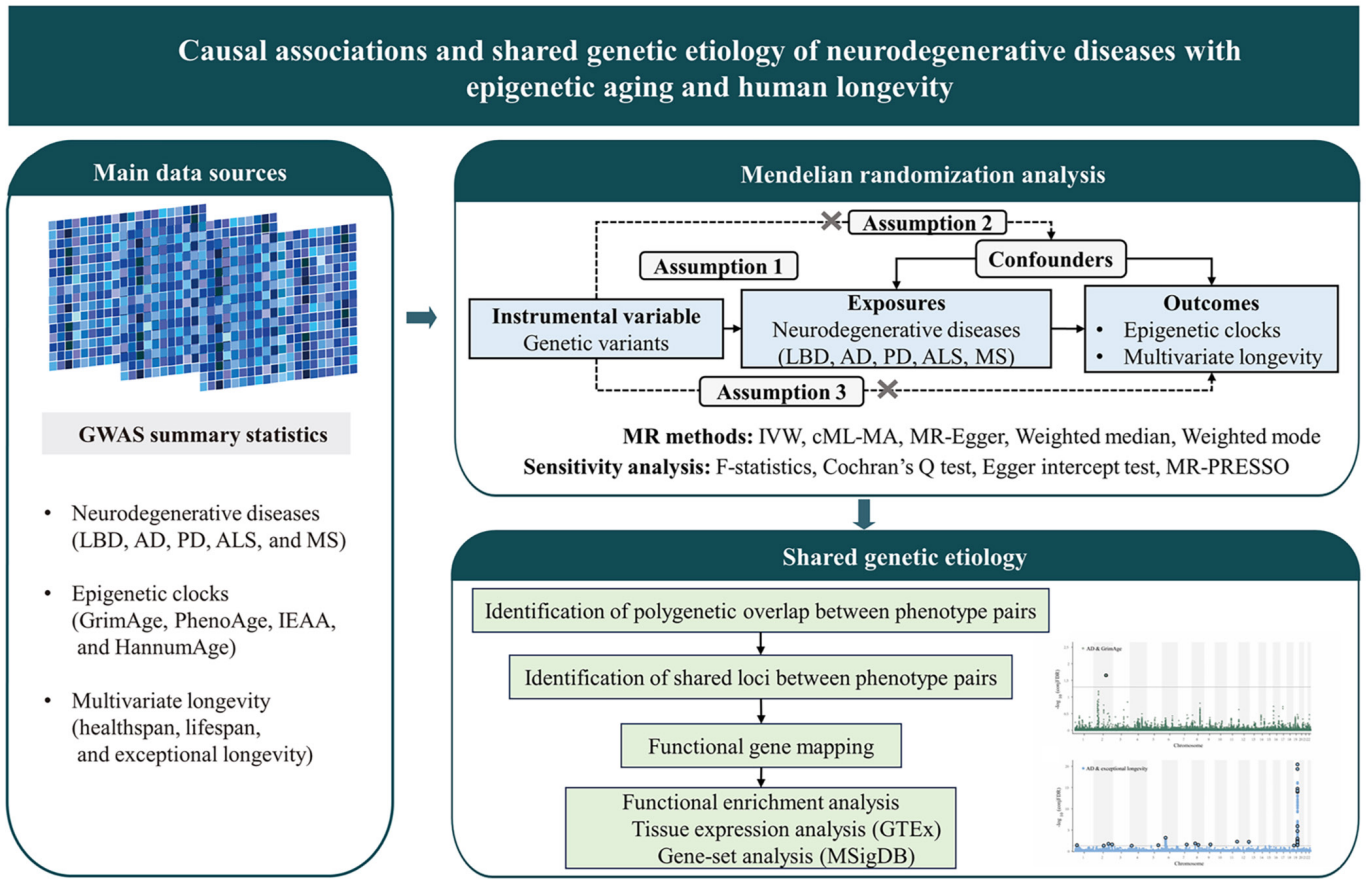
Study overview. An overview of this study's data sources and main analyses. AD, Alzheimer's disease; ALS, amyotrophic lateral sclerosis; cML‐MA, constrained maximum likelihood and model average‐based MR method; GTEx, Genotype‐tissue Expression Project; IVW, inverse variance weighted; LBD, Lewy body dementia; MR, Mendelian randomization; MR‐PRESSO, Mendelian Randomization Pleiotropy‐RESidual Sum and Outlier; MS, multiple sclerosis; MsigDB, Molecular signatures Database; PD, Parkinson's disease.

In this study, we aimed to detect causal association and overlap in the genetic etiology between multiple neurodegenerative diseases (AD, PD, LBD, ALS, and multiple sclerosis [MS]) and four epigenetic clocks (GrimAge, PhenoAge, IEAA, and HannumAge), as well as neurodegenerative diseases and multivariate longevity‐related phenotypes (parental lifespan, healthspan, and exceptional longevity) (Figure 1). Toward this goal, we mainly utilized MR and conditional/conjunctional false discovery rate (cond/conjFDR) methods using large‐scale genome‐wide association study (GWAS) datasets. Further, we identified pleiotropic genetic variants, genes, and biological pathways that comprised the shared molecular phenotype for neurodegenerative diseases, epigenetic aging, and multivariate longevity‐related phenotypes.

## 2 RESULTS

### 2.1 Mendelian randomization analysis

We identified five independent SNPs for LBD, 33 for AD, 26 for PD, 94 for MS, and seven for ALS (Table S1). All selected independent SNPs' *F*‐statistics were greater than 10 (Table S1). None of SNPs were dropped as palindromes. Further, these SNPs were employed for MR analysis.

**FIGURE 2:**
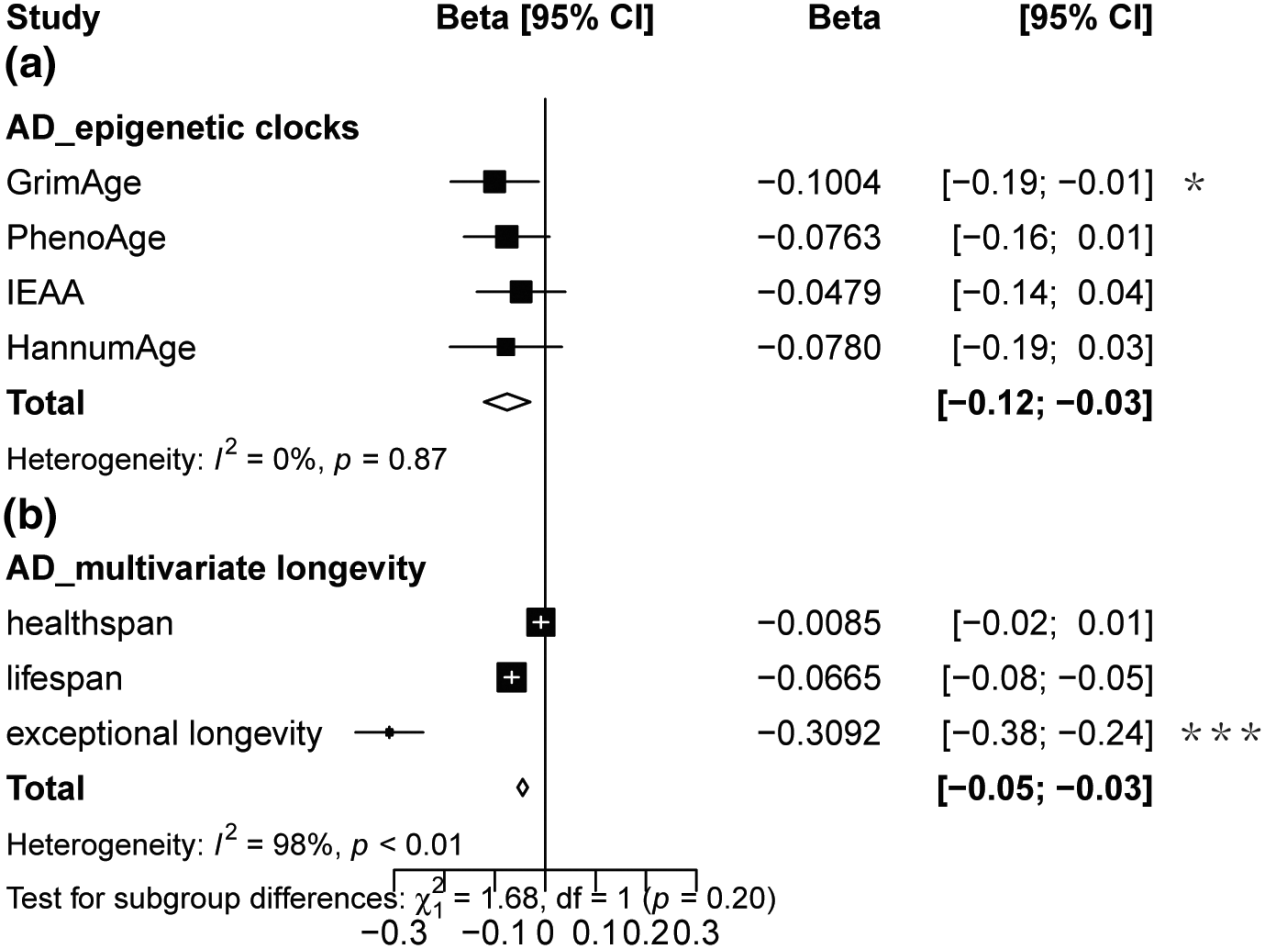
Results of inverse variance‐weighted Mendelian randomization analysis. Inverse variance‐weighted Mendelian randomization estimates for genetically predicted effects of Alzheimer's disease on epigenetic age accelerations (a) and multivariate longevity (b). IEAA, Intrinsic HorvathAge. MR result of each neurodegenerative disease subgroup was pooled by fixed effect meta‐analysis. *Indicated suggestive significance. ***Indicated significance.

Significant evidence showed that AD patients had 0.309 year less in exceptional longevity (IVW beta = −0.309, 95% CI −0.38 to −0.24, *p* = 1.51E‐19 < 3.33E‐03) (Figure 2, Table S2). As a complementary method for IVW method, constrained maximum likelihood and model average‐based MR (cML‐MA) method also indicated significant causal effect (beta = −0.06, 95% CI −0.07 to −0.05, *p* = 3.31E‐20 < 3.33E‐03) (Table S3). These *p*‐values passed multiple correcting tests. Next, we observed suggestively significant causal evidence between AD and GrimAge age acceleration (IVW beta = −0.10, 95% CI −0.188 to −0.013, *p* = 0.02; cML‐MA beta = −0.04, 95% CI −0.159 to −0.0002, *p* = 0.04) (Figure 2, Tables S3 and S4). The directions of sensitivity analyses (weighted median, simple mode, and weighted mode) were the same as the estimated effects of the main analysis.

In addition, we did not find any robust causal effects of other neurodegenerative diseases on epigenetic aging and human longevity (Figures S1 and S2, Tables S2–S4).

### 2.2 Sensitivity analysis

To ensure the suggestive and significant causal associations illustrated above were robust, we further performed sensitivity analysis. Multiplicative random effect IVW method we performed could reduce the bias caused by the heterogeneity for more accurate estimation of causal effects (Bowden et al., 2017; Higgins et al., 2003). To further detect whether heterogeneity exists, Cochran's *Q* test suggested no heterogeneity of heterogeneity of AD on GrimAge age acceleration (*p* = 0.60) and exceptional longevity (*p* = 0.45) (Table S5). We did not find horizontal pleiotropy between AD and GrimAge age acceleration (MR‐PRESSO causal estimate = −0.10, *p* = 0.69). Moreover, there was no horizontal pleiotropy between AD and exceptional longevity (MR‐PRESSO causal estimate = −0.31, *p* = 0.17) based on MR‐PRESSO analysis (Table S6). Overall, sensitivity analyses indicated that the MR results of AD on GrimAge age acceleration, and AD on exceptional longevity were robust.

### 2.3 Cross‐trait enrichment

**FIGURE 3:**
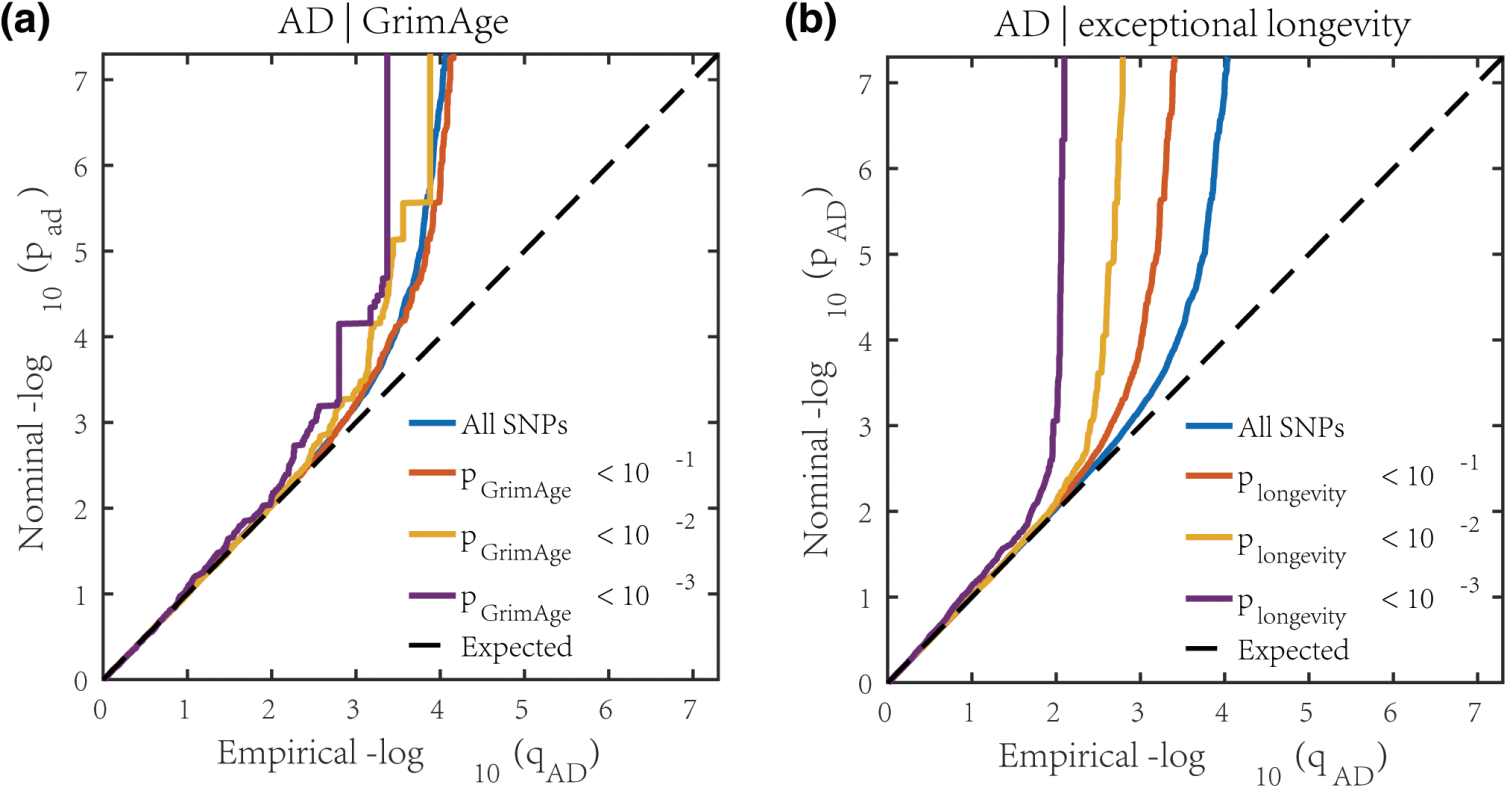
Cross‐trait enrichment between Alzheimer's disease, epigenetic aging, and human longevity. Quantile‐quantile (Q‐Q) plots illustrate cross‐trait enrichment between AD and GrimAge age acceleration (a), as well as AD and exceptional longevity (b). Conditional Q‐Q plots of nominal versus empirical $-\log_{10}p$, in which *p* represents the *p* values corrected for inflation, in primary phenotypes below the GWAS significance threshold of *p* < 5 × 10^−8^ as a function of significance of association with the second phenotypes, at *p* < 0.10, *p* < 0.01, *p* < 0.001. The dashed lines indicate the null hypothesis. The blue lines indicate all SNPs.

The conditional Q‐Q plots illustrated successive increments of SNPs enrichment for AD as a function of significant associations with GrimAge (Figure 3a) and exceptional longevity (Figure 3b), suggesting polygenic overlap. The enrichment results of reverse conditional Q‐Q plots were consistent with those of the forward conditional Q‐Q plot (Figure S3).

### 2.4 Shared loci between Alzheimer's disease, epigenetic aging, and human longevity

**FIGURE 4:**
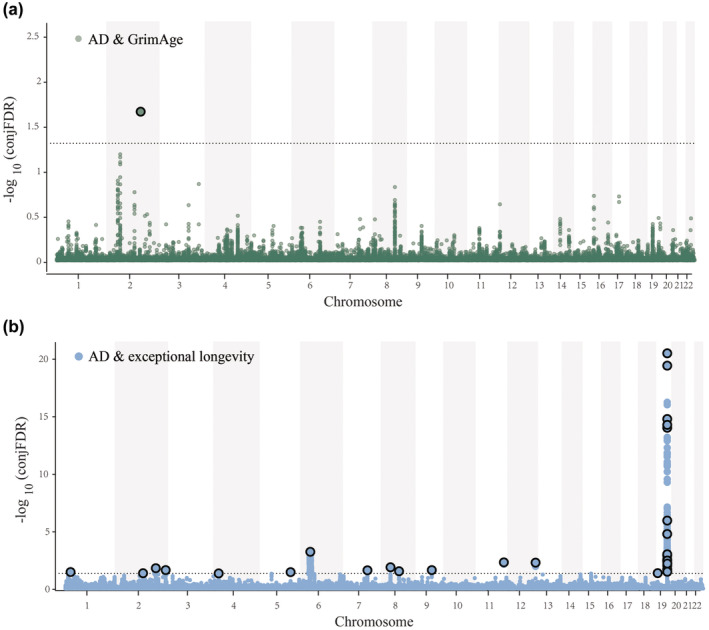
Shared loci between Alzheimer's disease, epigenetic aging, and human longevity. Common genetic variants jointly associated with AD and GrimAge age acceleration (a), and AD and exceptional longevity (b) at conjFDR < 0.05. Manhattan plots showing the −log10 transformed conjFDR values for each SNP on the y‐axis and chromosomal positions along the *x*‐axis. The dotted horizontal lines represent the threshold for significant shared associations (conjFDR < 0.05, i.e., $-\log10\left(\text{conjFDR}\right)>1.3$). Independent lead SNPs are circled in black. More detailed information about the identified loci, please see Tables S8 and S10.

Following the determination of the presence of polygenic overlap, we performed bidirectional cross‐trait enrichment to increase statistical power, and utilized conjFDR analysis to detect shared genomic loci. We identified one distinct genomic locus (i.e., rs78143120) shared between AD and GrimAge age acceleration (Figure 4a, Table 2, Table S8). Colocalization analysis also indicated rs78143120 (PP.H4 = 0.82) was the locus shared with both traits (Table S9). This locus on chromosome 2 at AC093375.1 was novel for AD. Besides, 14 distinct genomic loci associated with AD and exceptional longevity were identified by conjFDR, nine of which were defined as novel loci (Figure 4b, Table 2, Table S10). Among these loci, only rs12691088 (PP.H4 = 1) was the shared locus detected by colocalization analysis (Table S9).

Subsequently, we assessed the effect directions of the lead SNP at each shared genomic locus (Tables S8 and S10). Eight of the 14 shared lead SNPs exhibited consistent effect directions between AD and exceptional longevity. Besides, the effect direction of the lead SNP shared between AD and GrimAge age acceleration was inconsistent.

### 2.5 Functional annotation

Functional annotation of candidate SNPs jointly associated with AD and GrimAge age acceleration illustrated that they were all in intergenic regions (Table S11). Ten candidate SNPs associated with AD and exceptional longevity were functionally annotated to exonic region (Table S12). Missense mutations were found in seven of the 10 candidate SNPs (Table S13). Besides, 27 loci shared between AD and exceptional longevity had CADD > 12.37, indicating potentially detrimental effect (Table S12).

Further, we mapped seven, and 191 protein‐coding genes to candidate SNPs jointly associated with AD and GrimAge age acceleration, as well as AD and exceptional longevity, respectively (Tables S14 and S15). Some genes mapped to the loci associated with AD and GrimAge age acceleration, as well as AD and exceptional longevity were linked to neurodegenerative disorders, such as KCNJ3 and ERMN were associated with epilepsy (Bartnik et al., 2012; Leitner et al., 2022); NR4A2 involved in intellectual developmental disorder with language impairment and early‐onset dopa‐responsive dystonia‐parkinsonism (Kochmanski et al., 2022); APOE, known as the genetic risk factor for AD (Guo et al., 2023).

Finally, we identified 16 gene‐sets that exhibited significant enrichment with the genes mapped to the loci shared between AD and exceptional longevity (Table S16). The most significant enrichment gene‐sets were very low‐density lipoprotein particle clearance for gene ontology (GO) biological process, and protein–lipid complex for GO cellular component. The gene‐set analysis of genes mapped to shared loci between AD and GrimAge age acceleration was not available due to the poor mapped gene numbers.

## 3 DISCUSSION

To the best of our knowledge, this study is the first to investigate the causal associations and shared genetic etiology between neurodegenerative diseases and epigenetic age acceleration, as well as neurodegenerative diseases and multivariate longevity. Evidence indicated AD was causally associated with GrimAge age acceleration and exceptional longevity. Following the discovery of polygenic overlap, we identified one shared genomic locus between AD and GrimAge age acceleration, as well as one for AD and exceptional longevity. Among these distinct loci identified, only one novel for AD. Interestingly, we found some significantly enriched gene‐sets by Gene Ontology gene‐set analysis of genes mapped to shared loci between AD and exceptional longevity, which were all associated with synthesis and clearance of lipoproteins.

Epigenetic clocks have been applied in AD, PD, and ALS, but studies of epigenetic clocks in LBD and MS are still limited. A cross‐sectional study suggested that epigenetic age was positively associated with some AD risk factors, such as BMI, total cholesterol to high‐density lipoprotein cholesterol ratios, etc. (McCartney et al., 2018). McCartney et al. revealed that epigenetic age was younger 20 years than chronological age in 11 patients with late‐onset AD patients (LOAD) (Coto‐Vilchez et al., 2021). This direction of epigenetic acceleration is consistent with our results. However, the years of epigenetic age deceleration examined by McCartney et al. were quite different from our study. The following three reasons may explain this: (1) the degree of epigenetic acceleration varies between different age groups. The age of the LOAD samples in the study by McCartney et al. was about 90 years, which is older than the AD samples in our study; (2) AD subtypes (i.e., LOAD and AD) differ in epigenetic acceleration; and (3) different epigenetic clocks may predict different epigenetic ages. McCartney et al. used the Horvath Clock in their study, while ours was the GrimAge, and they may capture different characteristics of aging. Besides, studies suggested that epigenetic clock acceleration was linked to age at onset of PD (Tang et al., 2022; Xu et al., 2024) and age at onset and prognosis of ALS (Zhang et al., 2016, 2017, 2020).

Philip et al. have shown that dementia is associated with reduced life expectancy (Dumurgier & Sabia, 2021). AD is the most common dementia, accounting for 60%–80% of all dementia cases (Dumurgier & Sabia, 2021). A 6‐year follow‐up study showed that the survival rate of people with AD was significantly shorter both after the onset of symptoms and after diagnosis (Gerritsen et al., 2019). Totally, our study also found that AD reduced life expectancy, which was consistent with the findings of previous studies.

Some may assume that the inconsistent causal effects of AD on the four measures of epigenetic age suggest that the causal relationships between them are uncertain and not plausible. It should be noted that this was not, in fact, a contradiction in terms. This was due to the fact that computational models of different epigenetic clocks were based on different tissues, DNA methylation sites and methods trained to capture different features (Horvath & Raj, 2018).

Some of distinct loci identified by cond/conjFDR associated with AD and exceptional longevity were significant, which was inconsistent with the results of the colocalization analysis. We suggest that the differences found between methods may also imply that these loci are associated with both traits. For example, although the four distinct loci (rs11694743, rs35499311, rs11777131, and rs9665907) identified as shared loci between AD and exceptional longevity only by cond/conjFDR method were not detected by colocalization analysis, these four loci have been shown to be associated with both phenotypes (Kunkle et al., 2019). Therefore, to be more robust, we only considered that the loci (rs78143120 and rs12691088) identified by both two methods were shared distinct loci between traits. For the other 13 loci, we cautiously assumed that they might be the shared loci between AD and exceptional longevity, and more experiment are needed to confirm.

Among the above 15 distinct loci, 10 of them were defined as novel loci in AD. Among these novel loci, rs78143120 was mapped to AC093375.1. A cross‐trait meta‐analysis revealed that rs61597598 (AC093375.1) may be associated with snoring and AD (Mahajan et al., 2022). Further experimental evidence is needed to confirm whether rs78143120 is strongly associated with AD. rs1067221 was mapped to ECE1 (endothelin converting enzymes 1), which could degrade amyloid‐β (Aβ) (Calabrese et al., 2022; Wang et al., 2023). rs4436949 was mapped to *INPP5D*, the expression of which has been reported to be associated with AD risk and induced by plaque‐associated microglia. rs4150196 was significantly associated with intelligence (Savage et al., 2018; Williams et al., 2023). It is well known that high intelligence and cognitive ability reduce the risk of AD, which supports the reliability of our analyses. rs10265385 was mapped to *ZNF655*, which was involved in transcriptional regulation in the pathogenesis of AD (Bis et al., 2020). rs913807 was mapped to *SEMA4D*, which was a key factor in central nervous system diseases. rs9665907 was mapped to *SCARB1* (Aβ‐receptors Scavenger receptor B1). Reduced SCARB‐1 protein expression increases Aβ plaque deposition, but has no effect on microglial accumulation around Aβ plaques, and actually worsens cognitive deficits in learning and memory (Wilkinson & El Khoury, 2012). rs12185519 was mapped to *KDM4B*, which has the potential to inhibit brain diseases such as AD by blocking *ICAM1* and *VCAM1*‐induced extravasation (Choi et al., 2017). These novel loci are all associated with pathways or causes of neurological disorders. Further experiments and researches are needed to confirm it.

This study has some limitations. First, individual‐level data were unavailable. Therefore, we could not perform a more detailed analysis of patients with neurodegenerative diseases in different age groups. Second, to minimize bias arising from population stratification, only individuals of European ancestry were included in this study. Further research is required to confirm our results in other populations. Third, EAA or deacceleration may occur in participants with neurodegenerative diseases, which may bias the result of genetic overlap. Nevertheless, this potential bias could not account for the mixed patterns of effect directions among shared loci. Finally, given the paucity of previous genetic evidence about some novel loci we identified, we considered that this result should be interpreted with caution and needed more experimental studies to validate.

In conclusion, this study demonstrated the causal associations and shared genetic etiology of neurodegenerative diseases with epigenetic aging and human longevity. We identified two shared risk loci exhibiting mixed effect directions, of which one was defined as novel risk locus for AD. Nevertheless, further biological evidence is required to reveal the underlying mechanisms.

## 4 MATERIALS AND METHODS

### 4.1 Neurodegenerative diseases GWAS dataset

We obtained the publicly available GWAS summary statistics of five neurodegenerative diseases (LBD, AD, PD, ALS, and MS). Summary statistics of SNPs associated with LBD were derived from the published genome‐wide analysis (Chia et al., 2021), which consisted of 2591 cases with clinically diagnosed LBD and 4027 controls.We obtained AD, PD, ALS, and MS summary‐level data from the International Genomics of Alzheimer's Project (IGAP) (Kunkle et al., 2019), the International Parkinson's Disease Genomics Consortium (IPDGC) (Nalls et al., 2019), the ALS Variant Server (AVS) (Borgheai et al., 2024; Nicolas et al., 2018), and the International Multiple Sclerosis Genetics Consortium (IMSGC) (International Multiple Sclerosis Genetics, 2019), respectively (Table 1). For sample sizes and more information on each dataset, see Table 1. All participants in the above datasets were mainly of European ancestry.

**TABLE 1:** Detailed information of datasets in this study.

Phenotypes	Sample size (cases/controls)	Consortium	References
Epigenetic clocks	34,710	–	McCartney et al. (2021)
Multivariate longevity			
Healthspan	300,477	–	Zenin et al. (2019)
Parental lifespan	1,012,240	–	Timmers et al. (2019)
Exceptional longevity	36,745	–	Deelen et al. (2019)
Neurodegenerative diseases			
Lewy body dementia	2591/4027	–	Chia et al. (2021)
Alzheimer's disease	21,982/41,944	IGAP	Kunkle et al. (2019)
Parkinson's disease	33,674/449,056	IPDGC	Nalls et al. (2019)
Amyotrophic lateral sclerosis	20,806/59,804	AVS	Nicolas et al. (2018)
Multiple sclerosis	47,429/68,374	IMSGC	International Multiple Sclerosis Genetics (2019)

*Note*: All participants in the above datasets were mainly of European ancestry.

**TABLE 2:** Distinct loci associated with AD at conjFDR <0.05.

CHR	LEAD SNP	Nearest gene	A1/A2	*p*‐Value	Novel in AD	Shared phenotype
2	rs78143120	*AC093375.1*	T/C	5.85E‐05	Novel	GrimAge
1	rs1067221	*ECE1*	A/C	8.30E‐04	Novel	Exceptional longevity
2	rs11694743	*BIN1*	G/A	1.59E‐04	Kunkle et al. (2019)	Exceptional longevity
2	rs35499311	*RPL21P32*	G/A	4.79E‐06	Kunkle et al. (2019)	Exceptional longevity
2	rs4436949	*INPP5D*	G/A	5.11E‐04	Novel	Exceptional longevity
4	rs2643431	*RP11‐608B3.1*	G/A	1.10E‐03	Novel	Exceptional longevity
5	rs4150196	*HBEGF*	T/C	2.19E‐04	Novel	Exceptional longevity
7	rs10265385	*ZNF655:GS1‐259H13.10*	T/C	5.41E‐04	Novel	Exceptional longevity
8	rs11777131	*CLU*	T/C	3.13E‐04	Kunkle et al. (2019)	Exceptional longevity
8	rs2086538	*RP11‐326E22.1*	G/A	6.92E‐04	Novel	Exceptional longevity
9	rs913807	*SEMA4D*	A/C	5.59E‐04	Novel	Exceptional longevity
11	rs9665907	*SORL1*	A/G	2.30E‐05	Kunkle et al. (2019)	Exceptional longevity
12	rs10773111	*SCARB1*	T/C	7.56E‐05	Novel	Exceptional longevity
19	rs12185519	*KDM4B*	T/C	2.69E‐238	Novel	Exceptional longevity
19	rs12691088	*APOC1*	A/G	8.14E‐04	Schwartzentruber et al. (2021)	Exceptional longevity

### 4.2 Epigenetic clocks GWAS dataset

We obtained four summary‐level datasets for epigenetic clocks (HannumAge, IEAA, PhenoAge, and GrimAge) (Table 1). Each dataset contains 34,710 participants of European ancestry (McCartney et al., 2021). Briefly, McCartney et al. evaluated and calculated age‐adjusted DNA methylation‐based measures of the four epigenetic clocks we previously mentioned, utilizing Horvath epigenetic age calculator software, an online tool (https://dnamage.genetics.ucla.edu/) or stand‐alone scripts from Steve Horvath and Ake Lu. Subsequently, outlier sample filtering, quality control, and imputing genotypes were performed. Imputation of genotypes were conducted using Haplotype Reference Consortium or 1000 genomes phase 3 panels. Sex and genetic principal components were adjusted by linear models. More detailed information on the research design and experimental procedures is available from McCartney et al. (2021).

### 4.3 Multivariate longevity GWAS dataset

With the aim of exploring the more clinically relevant phenotypes of aging and further analyzing the genetic associations between these phenotypes and neurodegenerative diseases, we obtained multivariate GWAS summary statistics from traits associated with human longevity (Timmers et al., 2020) ($N_{\text{total}}$ = 1,349,432, $N_{\text{effective}}$ = 709,709). The multivariate GWAS summary‐level dataset comprises four aging phenotypes (Timmers et al., 2020), which are healthspan (Zenin et al., 2019), parental lifespan (Timmers et al., 2019), and extreme longevity (Deelen et al., 2019) (Table 1). All participants in the datasets were of European ancestry.

### 4.4 Instrumental variables selection

Genetic instrumental variables of neurodegenerative diseases were identified by the genome‐wide significance (*p* < 5.00E‐08) and the linkage disequilibrium (LD) clumping cutoff (*r* ^2^ < 0.01). The threshold of the genome‐wide significance (*p* < 5.00E‐08) was adjusted by Bonferroni correction for the numbers of SNPs (Dudbridge & Gusnanto, 2008; Pe'er et al., 2008; Wellcome Trust Case Control Consortium, 2007). It has become the standard significance threshold in GWAS, especially for European population (Barsh et al., 2012; Pe'er et al., 2008). Palindromic SNPs were removed to avoid bias. *F*‐statistics is a measure of instrument strength in MR analysis. *F* > 10 indicates that the instrument has sufficient strength to estimate the causal effect reliably. We calculated *F*‐statistics by Equation (1) (*β*, effect size; SE, standard error) and considered instrumental variables with *F*‐statistics greater than 10 to be strongly associated with exposure (i.e., neurodegenerative diseases) (Burgess et al., 2011).

(1) $$F=\beta^{2}/{\mathrm{SE}}^{2}$$

### 4.5 Mendelian randomization analysis

MR analysis is an epidemiological method that utilizes genetic variants to provide statistical evidence for causality between the exposure (a risk factor) and outcome variables (Burgess et al., 2013; Hu, Zhang, Zhang, Gao, Wang, Wang, Han, International Genomics of Alzheimer's, et al., 2022; Hu, Zhang, Zhang, Gao, Wang, Wang, Han, Sun, et al., 2022). MR analysis requires the following three assumptions in order to ensure its results are robust. First, genetic variants (i.e., instrumental variables) should be significantly associated with the exposure (e.g., neurodegenerative diseases), with a genome‐wide significant level typically of 5.00E‐08. Second, instrumental variables should be independent of confounders of the exposure (neurodegenerative diseases). Third, instrumental variables should only have effects on the outcome variables (epigenetic clocks and multivariate longevity) by the exposure. The latter two assumptions are collectively referred to as independence from pleiotropy (Davies et al., 2018; Didelez & Sheehan, 2007).

The main MR analysis method was multiplicative random effects inverse‐variance‐weighted (IVW) MR. IVW MR incorporates the genetic prediction of the effects of neurodegenerative diseases on EAA and multivariate longevity through instrumental variants (Burgess et al., 2013). Bonferroni correction was applied for multiple testing of the main IVW results. The significance thresholds for multiple test correction were calculated as $p=0.05/\left(N_{\text{exposure}}\times N_{\text{outcome}}\right)$, where $N_{\text{exposure}}$ and $N_{\text{outcome}}$ are the number of exposure variables and outcome variables, respectively. $N_{\text{exposure}}\times N_{\text{outcome}}$ is the number of tests performed. Therefore, Bonferroni‐corrected significance thresholds for epigenetic clocks and multivariate longevity as an outcome are $p=\frac{0.05}{5\times 4}=2.50E‐03$ and $p=\frac{0.05}{5\times 3}\approx 3.33E‐03$, respectively. The main MR analysis was completed by “TwoSampleMR” R package. Additionally, we pooled MR results across neurodegenerative diseases using fixed‐effect meta‐analysis by R package “meta.”

We also performed a constrained maximum likelihood and model average‐based MR method (MR‐cML‐MA) as secondary MR analysis to control correlated and uncorrelated pleiotropic effects using the MRcML R package (Schwartzman & Lin, 2011; Xue et al., 2021).

### 4.6 Sensitivity analysis

We performed MR‐Egger, weighted median, and weighted mode methods as sensitivity analyses to determine how reliable IVW MR effect estimates were. MR‐PRESSO was utilized to identify outliers and estimate potential horizontal pleiotropy using “MR‐PRESSO” R package (Verbanck et al., 2018).

### 4.7 False discovery rate and colocalization analysis

We conducted condFDR and conjFDR methods to improve the discovery of specific genomic loci jointly associated with phenotypes that have strong causal relationships (Bahrami et al., 2020). CondFDR statistical framework builds on the standard FDR and incorporates the genetic summary statistics from the primary trait of interest (e.g., LBD) and those of a conditional trait (e.g., GrimAge age acceleration) to readjust the test statistics in the primary phenotype. Genetic variants with condFDR < 0.01 were considered as associated with the primary phenotype. ConjFDR is an evolution of condFDR, which applies cross‐trait enrichment between two traits to increase genetic discovery. Genetic variants with conjFDR < 0.05 were considered as shared loci (Andreassen et al., 2013). Subsequently, significant genetic variants identified were clustered into LD blocks at the LD *r* ^2^ > 0.1 based on 1000 Genome Project LD structure. Besides, we performed conditional quantile‐quantile (Q‐Q) plots to estimate pleiotropic enrichment and visualize the cross‐trait enrichment. Q‐Q plots illustrated the *p*‐values distribution of the primary phenotypes (e.g., LBD) for all SNPs, as well as the *p*‐values distribution of the SNP strata determined by their association with a secondary phenotype (e.g., GrimAge age acceleration). Considering the complex and unusual LD patterns and genetic architecture of the MHC region, we excluded SNPs around the extended major histocompatibility complex (MHC) region (chr6:25119106–33854733), chromosome 8p23.1 (chr8:7200000–12500000), and MAPT region (chr17: 40000000–47000000) based on human genome 19 locations before fitting condFDR and conjFDR model to avoid bias (Schwartzman & Lin, 2011). These analyses were completed by pleiofdr (https://github.com/precimed/pleiofdr).

We also employed colocalization analysis to detect whether the distinct shared loci identified by cond/conjFDR method were responsible for both traits (Giambartolomei et al., 2014). Based on a Bayesian algorithm, the colocalization method calculated posterior probabilities for five hypotheses. A locus was considered to be associated with both traits if the posterior probability of H4 was > 0.75. Colocalization analysis was performed by R package “coloc” with the default parameters.

### 4.8 Genomic loci definition

The definition of independent genomic loci was consistent with FUMA (Watanabe et al., 2017) protocol. Independent significant SNPs were considered as conjFDR < 0.05 and *r* ^2^ < 0.6. Further, the SNPs with *r* ^2^ < 0.1 among these SNPs were determined as lead SNPs. Candidate SNPs were identified by LD $r^{2}\geq 0.6$ with independent significant SNPs. If the distance between loci were < 250 kb (i.e., LD blocks < 250 kb apart), then they were merged. The SNP with the most significant conjFDR was selected as the lead SNP of the merged locus. The borders of the genomic loci were defined by identifying all candidate SNPs in LD at $r^{2}\geq 0.6$ with one of the independent significant SNPs in the locus. LD information was obtained and calculated by the 1000 Genomes Project reference panel (Genomes Project et al., 2015). The directional effects of the shared loci between phenotypes were evaluated by comparing their *z*‐scores and odds ratios. Novel loci were defined if it were not physically overlap with the reported loci in the original GWAS (±500 kb) or were not reported in NHGRI‐EBI catalog (MacArthur et al., 2017).

### 4.9 Functional annotation

We annotated SNPs using combined annotation dependent depletion scores (CADD) (Kircher et al., 2014), regulomeDB scores (Boyle et al., 2012), and chromatin states. CADD is a popular tool that uses machine learning methods to calculate a CADD score for each genomic loci to assess the degree of pathogenicity. RegulomeDB scores are RegulomeDB‐based scores that assess the potential functionality of each noncoding variant. We also utilized FUMA and Genotype Tissue Expression (GTEx) resources to achieve gene‐set enrichment and evaluate expression quantitative trait locus (eQTL) functionality.

## AUTHOR CONTRIBUTIONS

Y.G. designed research; Y.G. conducted research; Y.G. Y.K.W., and T.Y.L. analyzed data and performed statistical analysis; Y.G. wrote the paper. Y.K.W. helped proofread the manuscript. Y.H. and T.Y.Z. had primary responsibility for the final content. All the authors read and approved the final manuscript.

## FUNDING INFORMATION

This work was supported by the National Natural Science Foundation of China (grant number 62371161).

## CONFLICT OF INTEREST STATEMENT

None declared.

## Supporting information


Figures S1–S3

Tables S1–S16

## Data Availability

All analyses were performed using publicly available data. Summary‐level statistics for four epigenetic clocks are available at https://datashare.ed.ac.uk/handle/10283/3645. Summary‐level statistics for exceptional longevity are available at https://www.longevitygenomics.org/downloads; parental lifespan, https://datashare.ed.ac.uk/handle/10283/3209; healthspan, http://ftp.ebi.ac.uk/pub/databases/gwas/summary_statistics/GCST007001‐GCST008000/GCST007406/. Summary‐level statistics for LBD are available at https://www.ebi.ac.uk/gwas/studies/GCST90001390. Other neurodegenerative diseases datasets were available at consortiums, respectively. The code used in this study will be available from the corresponding author upon reasonable request.
