# Supplementary material for: Causal associations and shared genetic etiology of neurodegenerative diseases with epigenetic aging and human longevity
Source: Aging Cell. 2024 Sep 19;23(11):e14271. doi: 10.1111/acel.14271 (PMC11561668; doi:10.1111/acel.14271)
Supplement: Supplementary file 1 — Figures S1–S3 [file ACEL-23-e14271-s002.docx]

Causal associations and shared genetic etiology of neurodegenerative diseases with epigenetic aging and human longevity

Yu Guo^1, #^, Guojuan Ma^2, #^, Yukai Wang^1^, Tingyan Lin^1^, Yang Hu^1, *^, Tianyi Zang^1, *^

^1^School of Computer Science and Technology, Harbin Institute of Technology, Harbin 150086, China

^2^ Beidahuang Industry Group General Hospital, 150088, China

**Conflict of Interest and Funding Disclosure:** All authors have no conflicts of interest.

^#^These authors contributed equally to this work.

***Correspondence**

Corresponding Author: Yang Hu, Tianyi Zang

Telephone: 86-15045666752

Mail address: Yikuang Street 2, Nangang District, Harbin, 150001, China

Email address: [huyang@hit.edu.cn](mailto:huyang@hit.edu.cn), [tianyi.zang@hit.edu.cn](mailto:tianyi.zang@hit.edu.cn)


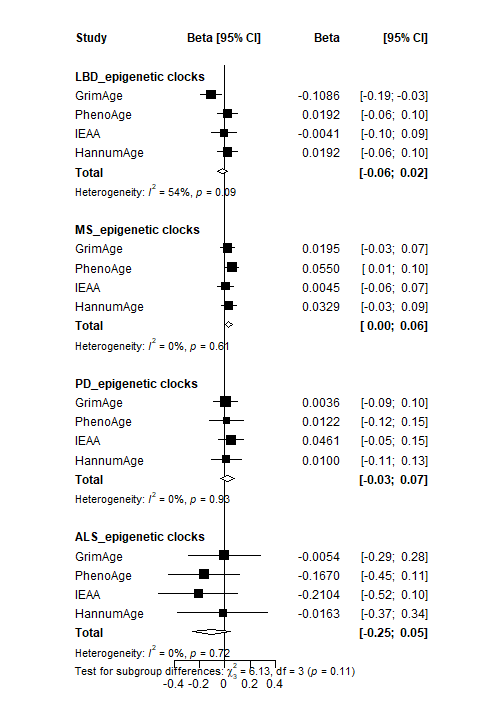
**Supplementary Figure 1.** **Inverse-variance weighted Mendelian randomization analysis results of neurodegenerative diseases on epigenetic age accelerations.**


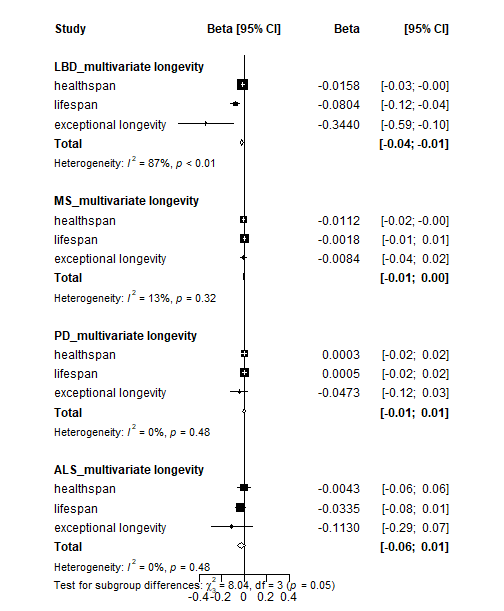


**Supplementary Figure 2. Inverse-variance weighted Mendelian randomization analysis results of neurodegenerative diseases on multivariate longevity.**


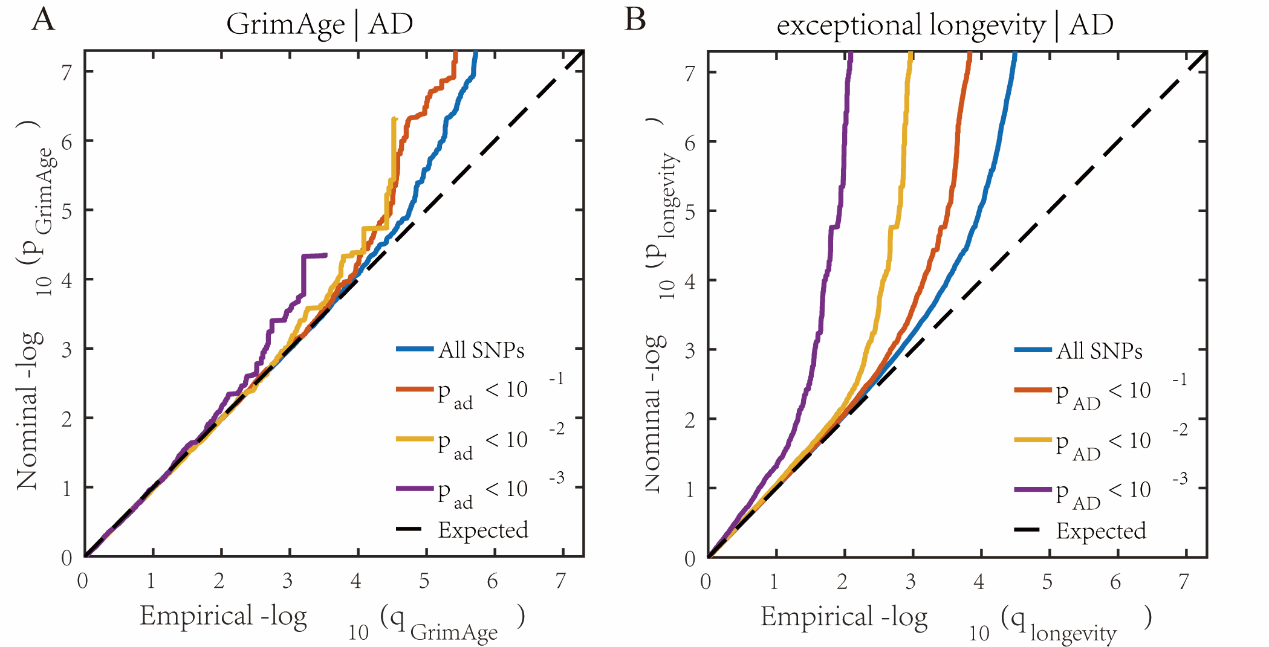


**Supplementary Figure 3. Reverse Cross-trait enrichment between** **Alzheimer’s disease, epigenetic aging and human longevity.** Quantile-quantile (Q-Q) plots illustrate cross-trait enrichment between GrimAge age acceleration and AD (A), as well as exceptional longevity and AD (B). Conditional Q-Q plots of nominal versus empirical $-{log}_{10}p$ , in which p represents the p values corrected for inflation, in primary phenotypes below the GWAS significance threshold of $p<5\times{10}^{-8}$ as a function of significance of association with the second phenotypes, at $p<0.10$, $p<0.01$, $p<0.001$. The dashed lines indicate the null hypothesis. The blue lines indicate all SNPs.
